# Cardiometabolic health profile of young girls with aesthetic professions

**DOI:** 10.1186/s12905-022-01599-z

**Published:** 2022-01-16

**Authors:** Salime Chedid Lisboa, Alexandra Vieira, Juliana Lopes Teodoro, Rochelle Costa, Franccesco Pinto Boeno, Juliano Farinha, Cláudia Gomes Bracht, Álvaro Reischak-Oliveira, Giovani dos Santos Cunha

**Affiliations:** 1 Universidade Federal do Rio Grande do Sul, Porto Alegre, Rio Grande do Sul, Brazil; 2 Faculdade Sogipa, Porto Alegre, Rio Grande do Sul, Brazil

## Abstract

**Background:**

In the literature, professions that impose body standards for daily performance are designated as non-conventional professions (i.e. models, athletes, ballet dancers), with great emphasis on the female population. More than a job, it becomes a lifestyle to those inserted in this environment, thus, thousands of children and adolescents seek inclusion and success in these professions due to financial and media gains. Such professions are associated with several health-related risk factors. The purpose of this study was to identify and compare among physical fitness levels, cardiometabolic health markers, mental health and dietary habits in non-conventional professions.

**Methods:**

The sample consisted of 41 female individuals aged between 14 and 24 years, allocated into four groups, control group composed by university students (UG = 11), models (MG = 11), ballet dancers (BG = 11), and athletes’ group (AG = 8). Physical fitness outcomes (cardiorespiratory fitness, flexibility, maximal dynamic strength, muscular endurance and body composition); biochemical outcomes (high-density lipoprotein [HDL], low-density lipoprotein [LDL], total cholesterol [TC], fasting glucose [FG], fasting insulin [FI], C-reactive protein [CRP]), diet quality and mental health were evaluated.

**Results:**

No impairments were observed in the health markers evaluated among groups, both for health-related physical fitness and biochemical outcomes. However, low levels of bone mineral density (BMD) were observed. Even with statistically significant differences between the groups for chronological age (p = 0.002), menarche (p = 0.004), career length (p = 0.001), height (p = 0.001), body mass index (p = 0.018), waist-to-height ratio (p < 0.001), %Fat (p = 0.020), VO_2peak_ (p = 0.020), maximal dynamic strength of knee extensors (p = 0.031) and elbow flexors (p = 0,001) and flexibility (p < 0.001), all these values are within the normal range for health.

**Conclusion:**

The professions analyzed do not seem to interfere in the physical fitness and cardiometabolic health of the girls assessed. However, we identified that exposure to these profession can impair mental health (depressive symptoms in 100% of participants) and body composition (BMD 63% of participants).

## Introduction

Body appearance and the pursuit of beauty conception have been directly associated with health since the nineteenth century, and nowadays, with success. Beauty has been treated/discussed in society since ancient Greece, in the Ancient Olympic Games it was already possible to observe an incitement to body strength and beauty, with the ideology of body standards required by the society, which already exalted the benefits that the physical exercise practice brought to the body [2–4]. Over time, changes have occurred in these body standards, reflecting traditions and customs of specific historical moments. It is also possible to realize that when searching for standardized bodies, the society considers that beautiful bodies are strongly linked to health, with exploration and enhancement of the female public appearance [4]. Faced with that, some professions gained prominence in the world scenario, as a consequence of an unconventional daily life to a great part of the world population, using aesthetic standards and high levels of daily performance, thus formatting body measures to act in the professions of models, athletes and ballet dancers [6, 7]. These individuals who have daily occupations related to aesthetics (particularly related to their body shape) are disproportionately exposed to modifiable (frequent measurements of body parameters, weight and body shape control) and unmodifiable (height) daily charges [2].

The beginning of these professions primarily occurs in childhood, with wide dedication during adolescence (10–19 years), a transition period between phases characterized by physical, metabolic and mental changes [8], and also by the search for economic independence and social inclusion [9]. The groups of non-conventional professions are selected for their aesthetics, meeting previously established measurements and body composition standards (low body weight accompanied by thinness), together with high daily performance. Many young women adopt daily routines and practices that can be harmful to their health in order to achieve inclusion and stability in these environments [10–12]. Thus, the daily practices carried out by this public have been reported as behaviors that compromise health factors, including cardiometabolic, nutritional, physical and psychological risks due to the specific demands of each profession [13–17].

Health researchers recognize that some diseases and disorders are highly prevalent in occupational subgroups (compared to the general population) due to harmful exposure and daily vulnerability to maintain aesthetic standards. Moreover, it is undeniable that these professions are powerful and boost even more body standards and the media. The low body weight imposed in these professions can reduce fat percentage to levels below the normative values (12% -15% between 7–17 years old and 13–16% between 18–25 years old, for young women) [18, 19]. These reductions can lead to impairment of health, such as changes in the lipid and metabolic profile [20], changes in the nutritional profile (food restriction, purging behaviors) causing eating disorders (such as anorexia and bulimia nervosa) [13], psychological disorders [21], along with changes in the physical exercise practice profile, with cases of increased exercise intensities in order to improve movement technique or accelerate body weight loss, as well as, in other cases, abandonment of physical exercise/sports practice, often caused due to injuries [22], overtraining [23] or burnout syndrome, due to early specialization in the professions [16].

Although, on the one hand, these professions may present some health risk factors [24], on the other hand, studies with aesthetics-related professions show that despite the adopted behaviors, these groups have some protective factors to health [25]. The practice of physical activities and exercises has achieved greater prestige as therapeutic and preventive tools for health promotion. When correctly applied, it proves to be a bioregulator of health indicators, which can cause an important connection between health and body standards.

Currently, the number of young people with health problems arising from low levels of physical fitness and physical activity is alarming [26]. Thus, for pediatric populations, the physical activity level is a demonstrative component of general health as it corresponds to an individual’s ability to perform physical exercise satisfactorily, integrating the musculoskeletal, hematological, neurological, cardiorespiratory and endocrine systems [27]. With a high levels of physical activity and adequate physical fitness, it is possible avoid future chronic disorders (cardiovascular, endocrine, osteoarticular, neuromuscular and psychological diseases) and protect against early mortality and morbidity [27, 28]. In this sense, it appears that the physical fitness levels plays an important role in the performance of young people who have non-conventional professions [29], however, it is not yet clear whether the participation in these groups of professions is associated with unfavorable bodily changes. Therefore, the purpose of the present study was to identify and compare the physical fitness, cardiometabolic risk factors, quality of life and depressive symptoms between young people belonging to non-conventional professions.

## Materials and methods

### Study design

**Fig. 1:**
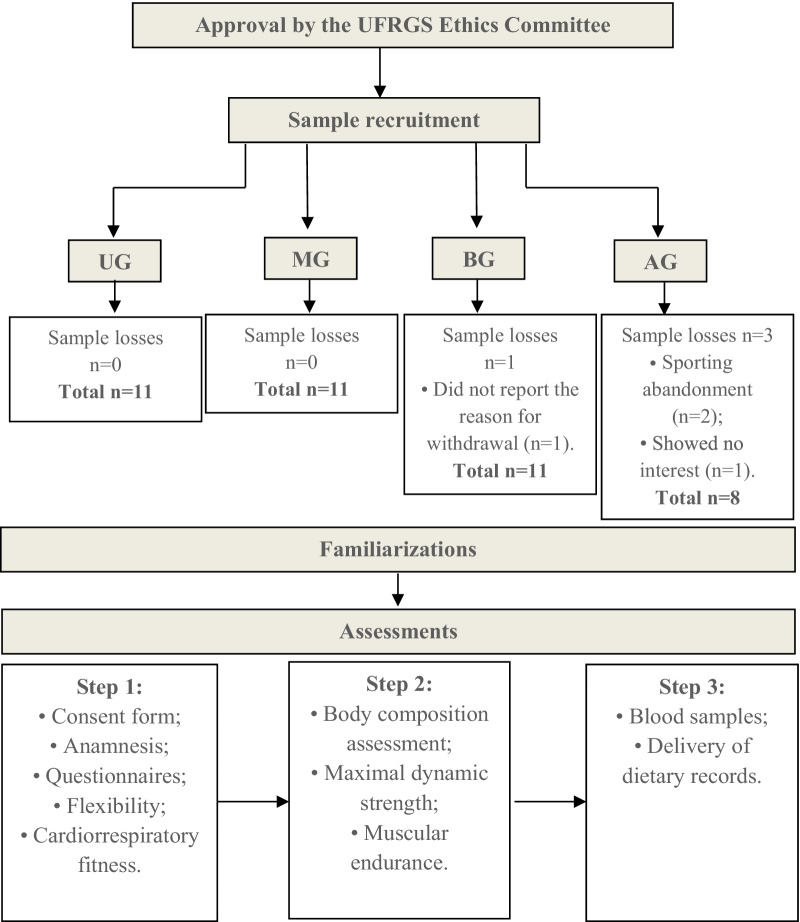
Flowchart of the experimental design of the study

The present study is characterized as cross-sectional, consisting of four groups. Of these, three were composed of non-conventional professions (models, athletes, and ballet dancers) and a control group (undergraduate students). All groups were submitted to the same assessment protocols (Fig. 1).

### Subjects

The sample consisted of 41 female teenagers and young adults aged 14 to 24 years who belonged to modeling agencies, sports teams, dance schools and universities from the city of Porto Alegre, Rio Grande do Sul, Brazil. The subjects were allocated according to their daily occupation: models’ group (MG = 11), ballet dancers’ group (BG = 11), athletes’ group (AG = 8) and undergraduate students’ group (UG = 11). Modeling agencies, sports clubs and dance schools also signed an assent form. The study was approved by the Research Ethics Committee of the University (CAAE: 67847317.5.0000.5347), and was conducted according to the standards proposed by the Helsinki Declaration [1]. The participants and their legal guardians (if participants are under 18 years old) were informed about the experimental protocol and the potential risks, providing written informed consent prior to participation (Fig. 1).

### Study overview

See Fig. 1.

### Body composition

Height (HEI) and body mass (BM) measurements were carried out. With these values, body mass index (BMI) was calculated (BMI = BM[kg]/HEI^2^[m]). In addition, bone mineral density (BDM), fat mass, fat-free mass (FFM) and fat percentage (%F) were evaluated in all participants (Lunar Densitometer model DPX.L, "Dual Energy X-ray Absorptiometry"). Data were processed by the system provided for each segment and the BDM results were expressed in g/cm^2^.

### Cardiopulmonary exercise testing (CPET)

Peak oxygen consumption (VO_2peak)_ was evaluated by open circuit ergospirometry during a maximum treadmill run test. The highest value reached in the last minute of exercise was used. According to the manufacturer’s instructions, the ergospirometry device (Quark CPET, Cosmed, Italy) was manually calibrated using the known concentrations of gases (21% O_2_ as reference concentrations, 12% O_2_ and 5.09% CO_2_ for calibration). The individuals had the possibility and time to adapt to the treadmill (Quinton Instruments, Seattle, USA) and the ergospirometry device. The participants performed a single progressive maximal exercise test, which consisted of walking for 4 min at 4 km h^−1^ followed by increases of 1 km h^−1^ at each minute until exhaustion. The individuals were verbally encouraged during the test to reach their maximal performance. To verify an exhausting effort, each participant had to meet at least one of the following criteria after the end of the test: (1) VO_2_ plateau that was defined as an increase in VO_2_ of less than 2.4 mL kg^−1^ min^−1^ with a corresponding increase in exercise intensity [30, 31], (2) obtainment of respiratory exchange ratio (RER) ≥ 1.0 [32, 33], (3) perception of effort greater than 17 (very intense—Borg’s scale of perceived exertion) [34]. Heart rate was monitored using a heart rate monitor (FT1–POLAR). In addition, the individuals demonstrated evident signs of extreme physical effort at the end of the test, such as facial flushing, sweating, hyperpnea and unstable gait [32, 33]. According to these criteria, VO_2peak_ of all participants were considered valid. All plots used in the determination of these points used breath by breath gross values. Two independent reviewers blindly determined VT and VCP following the criteria described above.

### One-repetition maximum and muscular endurance tests

Maximal strength was obtained by one-repetition maximum (1RM) test for bilateral knee extensors (KE) and bilateral elbow flexors (EF). Bilateral elbow flexion exercise was performed with barbells and dumbbells, whereas bilateral knee flexion exercise was performed in a strength training equipment (Konnen Gym). The subjects were familiarized with all procedures in a single session. On the test day, the participants carried out a warm-up for five minutes in a cycle ergometer, after having performed specific movements for the exercises of the tests. The 1RM was defined as the heaviest weight a participant could lift once with a proper lifting technique, without compensatory movements, with no more than five attempts with five minutes of recovery between attempts. After each successful performance, the weight increased until a failed attempt occurred [35, 36]. The rate of the gradual increase in load was dependent on the participant’s self-perceived capacity and readjusted for the next series using Lombardi [37] calculations. Performance time for each contraction (concentric and eccentric) was 1.5 s controlled by an electronic metronome (Quartz, CA, USA) [38]. After the performance of 1RM test, the load corresponding to 60% of 1RM of each individual was fixed. The subjects performed the maximum number of possible repetitions until fatigue. The number of repetitions performed was used as a measure of muscular endurance (ME) [39].

### Flexibility

To determine the flexibility of the hip, back and posterior muscles of the lower limbs, the sit and reach test was performed using the Wells bench. The subject remained barefoot, in a sitting position facing the equipment with the soles of the feet flat on the bench, stretched forward along the bench's metric demarcation, with outstretched arms and overlapping hands, seeking the greatest distance in three attempts, maintaining the value reached for 1 s. The highest value achieved was considered as a measure of flexibility [40].

### Blood analyses

For the performance of the biochemical analyses of the study, the subjects should be fasting for eight hours and performed the assessments between 48 and 72 h after the last exercise session [41]. Initially, a 30-min rest was performed, for further collection of 8 ml of a vein in the antecubital region. The samples were stored (vacuitaner tubes with EDTA) and centrifuged (3.500 rpm for 10 min), the plasma was aliquoted and frozen (-80°) for further analyses of fasting glucose (FG), fasting insulin (FI), total cholesterol (TC), triglycerides (TG), C-reactive protein (CRP) and high-density lipoprotein (HDL). Glucose levels were analyzed by enzymatic colorimetric method (Cobas C111, Roche, Diagnostics, Basel, Switzerland). Low density lipoprotein (LDL) was calculated by Friedewald formula (LDL:TC-HDL-TG/5) [42]. Plasma concentrations of FI were evaluated with commercial kits for humans (DRG International, Springfield, USA) by enzyme-linked immunosorbent assay (ELISA) according to the manufacturer’s instructions. For the lipid variables, the Brazilian Guidelines of dyslipidemias and atherosclerosis prevention [43] were used with determined health risk values for TC (< 170 mg/dL), HDL (> 45 mg/dL), LDL (< 110 mg/dL) and TG (< 90 mg/dL). Values lower than 100 mg/dL and between 1.9 and 23.0µUI/mL were used for FG and FI, respectively [44]. For the inflammatory profile, the value of <1 mg/L was used to determined values for CRP [32]*.*

### Dietary record

To evaluate dietary consumption and diet quality, a three-day dietary record was applied, describing daily dietary intake with the respective quantities. The record procedure was carried out as follows: each participant recorded all foods and beverages ingested in three days (two typical days and one atypical day of the weekend), also describing the eating schedule, quantities and when possible, the brand of the food. After filling in, all information was checked by a trained researcher, so that there would be no doubt about what was described. The dietary records were further calculated with the DietWin Professional Nutrition Software (Brubins CAS, Brazil).

### Depressive symptoms

Depressive symptoms assessment was carried out through the transcultural and self-applied CES-D depression scale, consisting of 20 statements on a Likert scale with four possible answers corresponding to number 1 (rarely or never – less than 1 day), 2 (few times – 1 to 2 days), 3 (a considerable time – 3 to 4 days) and 4 (all the time – 5 to 7 days). Four items of the instrument are presented with positive sense (blocking the trend to repetitive responses), such items are reversely scored (higher scores indicate greater amount of depressive symptoms). The cutoff score for Brazilian adolescents and young adults is 15, in which individuals with this score or higher are considered at risk for depression [45].

#### Statistical analysis

Normality of data distribution was evaluated by Shapiro Wilk test and homogeneity of variables was assessed by Levene test. Mean and standard deviation values were used for descriptive purposes. ANOVA one-way was used to analyze the differences between groups. LSD post hoc was used to establish the location of the differences between groups. Partial eta squared (η^2^) was calculated as a measure of effect size (ES). Values of 0.01, 0.06 and above 0.15 were considered small, medium, and high, respectively [46]. Kruskal–Wallis test was applied for the variables that did not present normal distribution. When the Kruskal–Wallis test was significant, pairwise comparisons were performed by Mann–Whitney U test for independent samples. Statistical analyses were performed with SPSS software (version 20.0, SPSS, Inc., IBM Company; NY, USA). The significance level adopted was p < 0.05.

### Results

Of the total sample, AG presented the youngest age (years) between groups (16.38 ± 3.02, p = 0.002), followed by BG (18.09 ± 3.21), MG (20.82 ± 2.52) and UG (21.91 ± 2.30). For career duration (months), the highest values were presented by BG (147.27 ± 65.97), followed by AG (132 ± 44.44) and MG (56.73 ± 36.04) (p = 0.001). Of the four groups, only three individuals of the AG had not yet gone through the first menarche (p = 0.004). For nutritional and psychological counseling, UG presented 5 individuals with nutritional counseling and 7 with psychological counseling, MG had 7 and 4, BG had 2 and 5 and AG presented 6 and 6, respectively.

Statistically significant differences between groups were found for height, BMI, WHtR, %Fat, fat mass, 1RM of KE and EF and flexibility (p < 0.05). In BMI, MG presented the lowest values (18.1 ± 1.37, p = 0.018) when compared to the other groups, however, it was within the expected for sex and age [47]. For WHtR, all groups presented lower values than the cutoff value for cardiovascular health [48]. In the 1RM of KE and EF, BG presented the lowest values in both limbs (1RM KE: 68.00 ± 16.89, p = 0.031; 1RM EF: 13.91 ± 2.39 p = 0.001). On the other hand, in the values obtained for flexibility, AG (50.38 ± 5.71 cm) and BG (45.77 ± 6.71 cm) presented significantly greater values compared to the other groups (UG: 36.41 ± 7.73; MG: 31.32 ± 9.96). For BMI, 14 individuals (34%) presented results below the normative values [49] for sex and age. As for flexibility, only 4 (10%) girls presented values considered bad [50] for sex and age. Even without statistically significant values, for BDM, 26 individuals (63%) showed results below the normative values [51, 52]. The results regarding body composition, cardiorespiratory fitness, maximal dynamic strength, muscular endurance and flexibility are presented in Table 1, whereas the individual responses are presented in Table 4.

**Table 1:** Physical fitness according to the profession

Variables	UG (n = 11)	MG (n = 11)	BG (n = 11)	AG (n = 8)	p value	η^2^
Body mass (kg)	58.16 ± 9.85	54.62 ± 4.52	51.94 ± 7.30	50.91 ± 5.35	0.278	0.07
Height (m)	1.66 ± 0.08^ab^	1.73 ± 0.02^a^	1.59 ± 0.06^ab^	1.60 ± 0.06^b^	0.001*	0.08
BMI (kg/m^2^)	20.9 ± 2.04^a^	18.1 ± 1.37^b^	20.5 ± 2.75^a^	19.8 ± 1.57^ab^	0.018*	0.23
WHtR	0.43 ± 0.01^a^	0.39 ± 0.02^b^	0.43 ± 0.04^a^	0.44 ± 0.02^a^	0.001*	0.44
%Fat	29.60 ± 4.29^a^	28.35 ± 4.12^a^	29.01 ± 4.81^a^	23.36 ± 4.20^b^	0.020*	0.23
Fat mass (kg)	16.46 ± 4.50	14.98 ± 2.61	14.64 ± 3.70	11.71 ± 3.07	0.069	0.07
FFM (kg)	40.20 ± 5.70	37.92 ± 3.62	35.74 ± 5.41	37.74 ± 2.76	0.187	0.12
BDM (g/cm^2^)	1.176 ± 0.115	1.122 ± 0.098	1.108 ± 0.073	1.132 ± 0.120	0.656	0.07
Vo_2peak_ (ml.kg^−1^.min^−1^)	45.13 ± 5.72^ab^	44.28 ± 3.90^b^	43.80 ± 3.97^b^	50.08 ± 3.62^a^	0.020*	0.23
1RM KE (kg)	90.18 ± 18.21^a^	78.09 ± 17.59^ab^	68.00 ± 16.89^b^	79.94 ± 11.73^ab^	0.031*	0.21
1RM EF (kg)	19.82 ± 3.49^a^	16.73 ± 2.28^a^	13.91 ± 2.39^b^	17.63 ± 2.00^a^	0.001*	0.08
ME KE (rep)	9.64 ± 2.11	10.09 ± 3.91	10.18 ± 2.44	10.25 ± 1.75	0.956	0.00
ME EF (rep)	9.00 ± 1.95	7.82 ± 2.32	6.82 ± 2.32	8.63 ± 3.02	0.104	0.07
Flexibility (cm)	36.41 ± 7.73^b^	31.32 ± 9.96^b^	45.77 ± 6.71^a^	50.38 ± 5.71^a^	0.001*	0.00

Data are presented as mean ± SD. Effect size eta squared (η^2^). Different letters indicate differences between groups

BMI: body mass index; WHtR: waist-to-height ratio; %Fat: fat percentage; FFM: fat–free mass; BDM: bone mineral density; Vo_2peak_: peak oxygen consumption; 1RM: one-repetition maximum; ME: muscular endurance; KE: knee extensors, EF: elbow flexors; rep: repetitions; UG: university students’ group; MG: models’ group; BG: ballet dancers’ group; AG: athletes’ group

*Indicates significant difference among groups (p < 0.05)

**Fig. 2:**
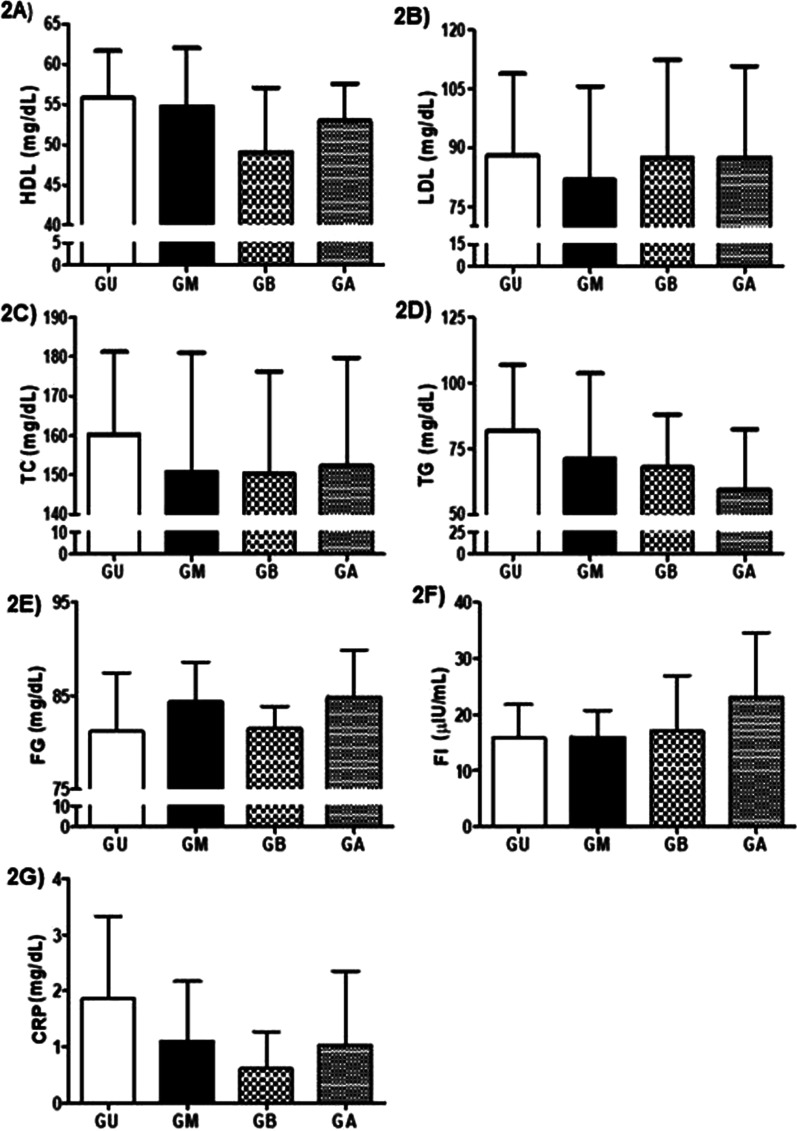
**a** High density lipoprotein (HDL); **b** low density lipoprotein (LDL); **c** total Cholesterol (TC); **d** triglycerides (TG); **e** fasting glucose (FG); **f** fasting insulin (FI); **g** C-reactive protein (CRP). UG: university students’ group; MG: models’ group; BG: ballet dancers’ group; AG: group. Data are presented as mean ± SD

When analyzing the lipid (HDL, LDL, TG, CT) inflammatory (CRP) and glycemic (FG and FI) variables, no statistically significant differences were found between groups (p > 0.05). The effect size values found were HDL: η^2^ = 0.14, LDL: η^2^ = 0.05, TC: η^2^ = 0.02, TG: η^2^ = 0.09, FG: η^2^ = 0.12, FI: η^2^ = 0.07, and CRP: η^2^ = 0.07 (Fig. 2). The individual responses are presented in Table 4. For HDL, only one individual (2% of the total) presented a non-normative value (> 45 mg/dL), for TC, 6 individuals (15%, < 170md/dL), for TG, 9 individuals (22%, < 90 mg/dL), for FI, 9 individuals (22%, from 1.9 to 230µUI/mL), and for CRP, 13 individuals (31%, < 1 mg/dL).

#### Dietary control

When analyzing the dietary control, no statistically significant differences were found between groups (p > 0.05). The mean energy intake (kcal) presented by AG is below the recommended (individually) for athletes who are exposed to the training loads described in this study (Table 2).

**Table 2:** Dietary control variables through the three-day dietary record for university students (UG), models (MG), ballet dancers (BG) and athletes (AG) in daily percentages (%)

	UG (n = 11)	MG (n = 11)	BG (n = 11)	AG (n = 8)	p value	η^2^
Mean energy intake (kcal)	2003.0 ± 658.9	1926.2 ± 686.7	1713.9 ± 460.4	1684.2 ± 459.0	0.731	0.09
Carbohydrates (%)	49.1 ± 4.5	49.8 ± 3.4	50.8 ± 6.8	52.0 ± 6.6	0.924	0.05
Proteins (%)	20.3 ± 3.3	20.8 ± 5.8	19.4 ± 3.4	19.6 ± 0.3	0.791	0.24
Lipids (%)	29.5 ± 3.5	28.1 ± 4.1	29.7 ± 4.9	28.2 ± 6.5	0.842	0.07

Data are presented as mean ± SD. Effect size eta squared (η^2^)

UG: university students’ group; MG: models’ group; BG: ballet dancers’ group; AG: athletes’ group

#### Depressive symptoms

The results regarding the depressive symptoms score are presented in Table 3. No statistically significant differences were found between groups (p > 0.05). All groups presented values above the cutoff points (> 15) for mental health. In addition, 53.6% of the girls were under psychological counseling. The individual responses are presented in Table 4. Of the total subjects of the study (41 individuals), 100% presented values that evidence a trend to depressive symptoms.

**Table 3:** General depressive symptoms values obtained through the CES-D questionnaire for university students (UG), models (MG), ballet dancers (BG) and athletes (AG)

	UG (n = 11)	MG (n = 11)	BG (n = 11)	AG (n = 8)	p value	η^2^
Depressive symptoms	39.00 ± 5.22	42.00 ± 7.07	41.60 ± 5.20	36.30 ± 4.66	0.127	0.13

Data are presented as mean ± SD. Effect size eta squared (η^2^)

UG: university students’ group; MG: models’ group; BG: ballet dancers’ group; AG: athletes’ group

**Table 4:**
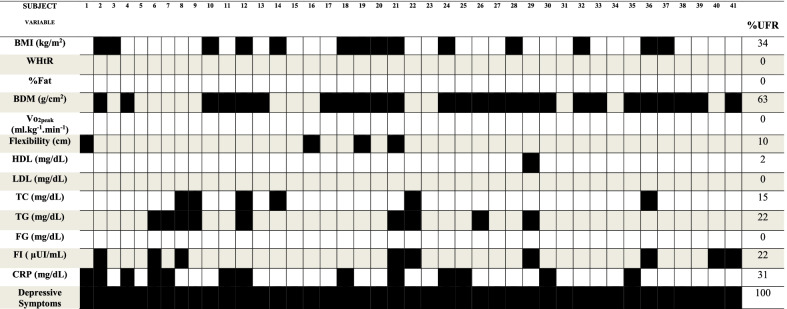
Individual behavior for the anthropometric, physical fitness, biochemical and depressive symptoms variables

BMI: body mass index; WHtR: waist-to-height ratio; %Fat: fat percentage; BDM: bone densitometry; Vo2peak: peak oxygen consumption; HDL: high-density lipoprotein; LDL: low-density lipoprotein; TC: total cholesterol; TG: triglycerides; FG: fasting glucose; FI: fasting insulin; CRP: C-reactive protein; %UFR- Percentage of unfavorable response. Black squares: present unfavorable response

## Discussion

The atmosphere of the professions evaluated in our study are considered to be highly competitive [24, 53, 54] due to the constant pressures on body shape [55]. However, even though the professions evaluated in our study are related to several health risk factors, the results did not identify impairments associated with their professional choice for cardiometabolic health indicators (VO_2peak_, body composition, WHtR, LDL, TC and FG) and dietary patterns. In this context, the individuals evaluated in our study demonstrated a current health status for these properties. However, we identified that exposure to there profession can impair mental health (depressive symptoms in 100% of participants) and body composition (BMD 63% of participants).

In Western society it is clear that being overweight is directly linked to unpleasant feelings that cause body dissatisfaction. Reports of female adolescents depict that thin women are more attractive and healthy [56]. However, such dissatisfaction with body weight and concerns to achieve an ideal beauty to fit in the body or professional framework are also associated with increased depressive symptoms [57]. For the mental health variable, all participants of our study presented results above the cutoff values for the Brazilian female population (> 15), corroborating previous studies that evaluated the same populations individually, which also found trend results for depressive symptoms [58–60]. In addition to the populations evaluated, there are evidences showing an increased prevalence of depressive disorders in the world population at increasingly earlier ages and twice as likely to occur in the female population [61]. During stressful situations, the human body suffers internal and external threats that cause changes and adaptations in both the physical and behavioral profiles. Physiologically, there is a stimulation of the sympathetic nervous system and the hypothalamus–pituitary–adrenal axis, and psychologically, high levels of stress can also develop depressive symptoms [62]. The individuals who comprised the sample of our study indicated that the demonstrated symptoms seem to be relatively common in the populations assessed, which is a warning sign, since the professions evaluated are the dreams of thousands of children and adolescents worldwide. The only group in our study that was not involved in any unconventional profession (UG) also demonstrated approximation of depressive symptoms in the same way as other groups. Adewuia et al. [63], in an epidemiological study with university students from different courses, found prevalence values between 15 and 25% of students with some type of manifestation of psychiatric disorders, with higher values for depressive disorders (prevalence of 8.3%). Such values are attributed to stressor agents throughout university courses, varying with the student's level (beginner, intermediate or advanced) [64]. Indeed, professional performance may not be markedly different for the prevalence of depressive symptoms to occur, according to the values found in our study [65].

Despite the daily particularities of the evaluated groups, an early professional initiation was observed in both groups of unconventional professions, with mean age of professional initiation at 19.5 ± 3.4 years and career lengths ranging from 4.7 years to 12.2 years, reaffirming that early professionalization seems to be unanimous for these professions. The highest values were found in BG and AG (the latter also presented the lowest mean age in our study, which is commonly found in gymnastics athletes) [66]. Along with this, 7.32% of AG participants had not yet gone through the first menarche. This result corroborates other studies in the area that evaluated late menstruation or amenorrhea in the same population [67], verifying that the age of first menarche for gymnastics athletes is delayed when compared to young people who do not exercise this modality, as well as aesthetic sports and modalities that demand a high level of performance, exposing young athletes to disorders at the level of the hypothalamic–pituitary–adrenal axis [68]. Young gynecological age, emotional or mental stress and rapid increases in training load (to achieve excellence in motor coordination, strength and flexibility) together with low body weight can influence the dysfunction at the beginning of the menstrual cycle [67].

The greater mortality risk for overweight individuals (situation defined by WHO as BMI 25.0 < 30.0 kg/m^2^) is widely exposed in the literature [42, 55, 69]. This occurs due to the increased risk of cardiovascular diseases resulting from negatives metabolic changes and health indicators, which increases the body’s oxidative stress (strongly linked to the obesity state), thus leading to a state of low-grade chronic inflammation in these individuals. The increase in adipose tissue leads to the production of pro-inflammatory cytokines that potentiate and endless cycle of inflammation and oxidative stress [70, 71]. However, discussions on metabolic health variations among individuals with BMI considered normative (18.5 < 25.0 kg/m^2^) are emerging [47, 72]. In our study, we evaluated these components in order to seek a deeper understanding about body composition distribution and cardiometabolic impact in girls involved in aesthetics-related professions. Cardiometabolic risk factors are due to biological changes (in lipid values, blood pressure, body weight, etc.) or related to lifestyle (inadequate diet, sedentary lifestyle, etc.). Analyzing the individual responses, 24.39% (10 individuals) of the participants in our study were metabolically unhealthy, a definition given by Stefan et al. [72] as a combination of at least two predisposing parameters for metabolic syndrome (UG: 5, MG: 4, BG: 2, AG: 1). In this regard, the scientific literature addresses that individuals with normative body weight who are not metabolically healthy have similar risks of mortality and cardiovascular events than metabolically healthy obese individuals [72, 73].

In relation to the results obtained for %Fat, of the total n of our study, 48.78% of the girls had nutritional monitoring and it is speculated that the lowest values found in the AG are due to the combination of training and diet monitoring, since most of the AG (75%) obtained nutritional counseling provided by the sports teams. In a previous study, Barlett et al. [74] found %Fat values around 16.8%, attributing the results to the aesthetic need of the sport as well as the balance between physical training and adequate nutrition.

Nonetheless, when verifying the mean energy intake (kcal) and the daily percentages of macronutrients indicated for adolescents [75], although the ingested values are considered adequate for all groups, the AG showed the lowest caloric intake of all groups. The Academy of Nutrition and Dietetics Dietitians of Canada [76] indicates that the daily caloric needs of athletes should be higher when compared to other groups, since the calculation of daily caloric needs must include the basal metabolic rate associated with the daily physical activity factor, in addition to the caloric expenditure promoted by physical exercise with estimated intensity in metabolic equivalents (Mets). It is therefore suggested that the daily diet of the athletes has mean daily values below what is required for the amount and intensity of physical exercise performed in training.

Due to the previously mentioned particularities about the professions evaluated in this study, the concern with the health of these populations gains greater relevance on a daily basis due to the difficulties faced in this age group and the historical daily pressure on the female body. Nowadays, healthy eating and physical exercise benefits are very widespread through the media, which may be assisting in the search for professionals for better day-to-day management of the professions approached. Therefore, we believe that the combination of good dietary intake along with physical exercise practice demonstrated by the groups may be helping the health status of the individuals in physical and metabolic parameters, contradicting the idea that these professions present a risk to the physical and metabolic health of adolescents.

## Conclusion

Based on the results found in our study, we observed that the professions analyzed and their aesthetic connection do not seem to interfere with the physical and metabolic health of the girls evaluated. However, all groups showed trends of decreasing mental health values. The comparison between groups showed great similarity in aspects of physical fitness and biochemical variables, regardless of whether the individuals had unconventional professions or not. However, even achieving satisfactory results, we believe that the populations involved need to be monitored daily since the age group (adolescence and young adults) is a period of formation and consolidation of life issues, in order to avoid future health problems. To our knowledge, this is the first study to evaluate the main professions considered at risk (models, ballet dancers and gymnasts) seeking to know the health indicators profile of these publics, comparing them with girls who do not work in unconventional professions.

## Data Availability

The datasets used and/or analyzed during the current study are available from the corresponding author on reasonable request.

## Abbreviations

MG: Models group
BG: Ballet dancers group
AG: Athletes group
UG: University students group
HEI: Height
BM: Body mass
BMI: Body mass index
BDM: Bone mineral density
FFM: Fat-free mass
%F: Fat percentage
CPET: Cardiopulmonary exercise test
VO_2peak_: Peak oxygen consumption
USA: United States
RER: Respiratory exchange ratio
1RM: One-repetition maximum
KE: Knee extensors
EF: Elbow flexors
ME: Muscular endurance
FG: Fasting glucose
FI: Fasting insulin
TC: Total cholesterol
TG: Triglycerides
CRP: C-reactive protein
HDL: High-density lipoprotein
LDL: Low-density lipoprotein
UG: Unconventional profession
Mets: Metabolic equivalents
